# Interactions between adenosine, angiotensin II and nitric oxide on the afferent arteriole influence sensitivity of the tubuloglomerular feedback

**DOI:** 10.3389/fphys.2013.00187

**Published:** 2013-07-18

**Authors:** A. E. G. Persson, En Yin Lai, Xiang Gao, Mattias Carlström, Andreas Patzak

**Affiliations:** ^1^Department of Medical Cell Biology, Uppsala UniversityUppsala, Sweden; ^2^Department of Physiology, Zhejiang University School of MedicineHangzhou, China; ^3^Department of Physiology and Pharmacology, Karolinska InstitutetStockholm, Sweden; ^4^Institute of Vegetative Physiology, Charité-Universitätsmedizin BerlinBerlin, Germany

**Keywords:** adenosine, angiotensin II, tubuloglomerular feedback, afferent arteriole, kidney

## Abstract

Adenosine, via activation of A_1_ receptors on the afferent arteriole (AA), mediates the tubuloglomerular feedback (TGF) mechanism. Angiotensin II and nitric oxide (NO) can modulate the sensitivity of the TGF mechanism. However, the interaction among these substances in regulating the TGF resetting phenomenon has been debated. Studies in isolated perfused AA have shown a biphasic response to accumulating doses of adenosine alone. In the nanomolar range adenosine has a weak contractile effect (7%), whereas vasodilatation is observed at high concentrations. However, a synergistic interaction between the contractile response by adenosine and that of angiotensin II has been demonstrated. Adenosine in low concentrations strongly enhances the response to angiotensin II. At the same time, angiotensin II in physiological concentrations increases significantly the contractile response to adenosine. Moreover, addition of a NO donor (spermine NONOate) to increase NO bioavailability abolished the contractile response from combined application of angiotensin II and adenosine. These mutual modulating effects of adenosine and angiotensin II, and the effect of NO on the response of AA can contribute to the resetting of the TGF sensitivity.

The tubuloglomerular feedback (TGF) is a negative-feedback system operating within the juxtaglomerular apparatus that can regulate glomerular filtration rate (GFR) by changing arteriolar resistance and hence blood flow and pressure into the glomerular capillaries. In this control system the tubular load to the distal parts of the nephron is detected via changes in tubular sodium chloride concentration at the macula densa site. This information is then used to determine the contractile state of the afferent arteriole (AA) that is the main effector link of this controller. The sensitivity and reactivity of the TGF system can be modulated via several different factors and via those changing the effector response. Exactly where and how this modulation of the TGF response occurs has not been clear. Recent work from our laboratory has indicated that this modulation to some extent can be carried out by the arterioles themselves.

Figure 1A shows signaling pathways of the TGF activated by an increase in NaCl delivery to the macula densa site. Evidence from our laboratory and others indicate that increased NaCl delivery leads to depolarization of the basolateral membrane of the macula densa cells, activation of nitric oxide synthases (NOS) to produce NO, and also activation of NADPH oxidase to produce superoxide (Persson et al., 1991; Liu et al., 2002; Liu and Persson, 2004). This activation of macula densa also leads to release of ATP, possibly via swelling of the macula densa cells that occur following increased uptake of NaCl (Gonzalez et al., 1988a,b).

**Figure 1:**
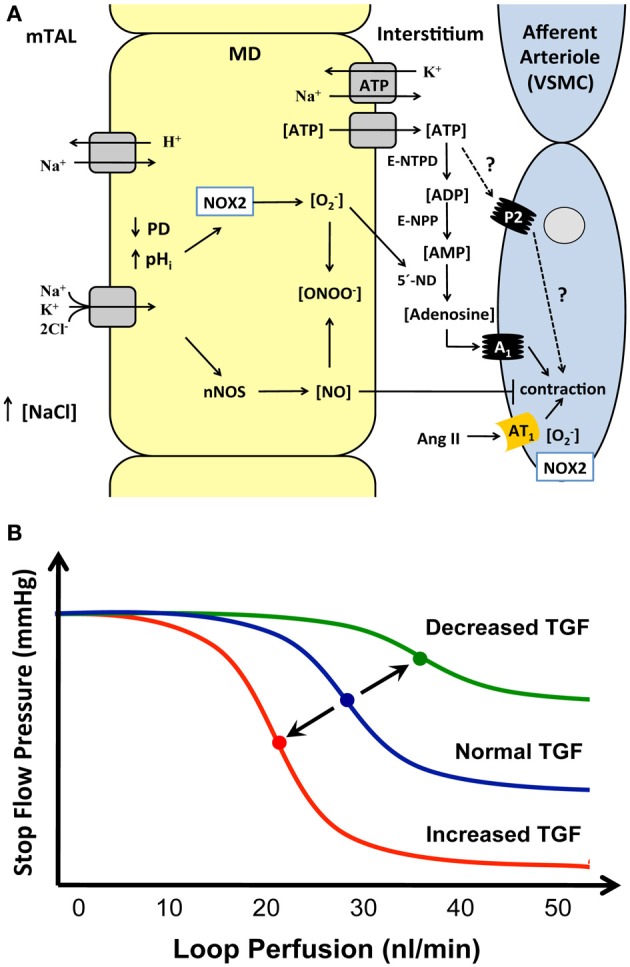
**(A)** Suggested mechanisms by which increased concentration of NaCl at the macula densa site increase apical Na/K/2Cl co-transporter and the Na/H–exchanger that will depolarize basolateral cell membrane potential which will activate NADPH oxidase 2 (NOX2) to increase production of O^−^_2_, but also increase NO production rate from nNOS. At the same time swelling of the macula densa cells can increase ATP leakage. ATP can then be broken down through ecto-5′-nucleotidase (5′-ND) and thereby increase the concentration of adenosine, this process can also be stimulated by O^−^_2_. The NO released could counteract the production of O^−^_2_ by forming peroxynitrite and in this way counteract the sensitization of the TGF. NO could also act directly on the AA to reduce the contractile response elicited by adenosine and angiotensin II (Ang II). Figure modified from that published in Carlstrom and Persson (2009). A_1_, adenosine receptor type 1; AT_1_, Angiotensin II receptor type 1; E-NPP, ecto-nucleotide pyrophosphatase/phospho-diesterases; E-NTPD, ecto-nucleoside triphosphate diphosphohydrolase; MD, macula densa; nNOS, neuronal nitric oxide synthase; NO, nitric oxide; NOX2, NADPH oxidase 2; P2, purinergic receptor type 2; PD, membrane potential; TAL, thick ascending limb of Henle's loop; VSMC, vascular smooth muscle cell. **(B)** The figure shows responses of the stop flow pressure to changes in the proximal tubular perfusion rate during renal micropuncture experiments. Middle curve demonstrates normal TGF response in normo-hydrated animals, the curve to the right shows a decreased TGF response with reduced sensitivity and activity and the curve to the left shows an increased TGF response with increased sensitivity and activity. A rightward shift can occur in situations with volume expansion or increased NO availability whereas a leftward shift can occur during dehydration, increased angiotensin II or ROS concentration or when NO levels are low.

Results from numerous studies reveal a crucial role of adenosine in the mediation of the TGF (Brown et al., 2001; Schnermann, 2002). Both adenosine A_1_ and A_2_ receptors are expressed on afferent arterioles, and can regulate preglomerular resistance. Adenosine in physiological concentrations constricts afferent arterioles via a prominent effect on purinergic A_1_-receptors (Hansen et al., 2003; Lai et al., 2006). The source for extracellular adenosine in the context of TGF is not fully cleared. Osswald and co-workers first proposed that local generation of adenosine in the juxtaglomerular apparatus may play an important role for signal transmission of TGF (Osswald et al., 1982). More recent experimental studies have demonstrated that ATP release from macula densa cells in response to increased NaCl load in the distal tubule (Bell et al., 2003). ATP itself has a constrictor effect of afferent arterioles via P_2_X receptors (Inscho et al., 1992), and therefore it has been debated weather ATP or adenosine is the mediator of TGF signaling. A direct role of ATP in mediating the TGF response may be possible, but the evidence for this is currently not compelling.

Mice lacking P_2_X_1_ receptors, which are present in vascular smooth muscle cells of afferent arteriole, display impaired preglomerular autoregulation (Inscho et al., 2003), but have largely normal TGF responses (Schnermann and Briggs, 2008). Furthermore, pharmacological inhibition of P2 receptors with suramin, did not significantly inhibit TGF of microperfused afferent arterioles with attached macula densas (Ren et al., 2004). ATP can be hydrolyzed by ecto-ATP diphosphohydrolases, the first step of extracellular degradation of nucleoside 5′-triphosphates and nucleoside 5′-diphosphates. Deficiency of NTPDase1/CD39 went along with impaired TGF responses in mice (Oppermann et al., 2008). Also, mice with ecto-5′-nucleotidase (cd73)-knock out showed decreased TGF responses (Castrop et al., 2004). This enzyme catalyzes the conversion of AMP to adenosine in the interstitium. The observations indicate an important role of extracellular degradation of ATP to adenosine in signaling of the TGF and for adenosine induced constriction of the arteriole. Although, ATP can constrict afferent arterioles, there is not much evidence for mediation of TGF by ATP. Rather, inhibition of P_2_X receptors did not influence TGF responses in acutely treated mice (Schnermann, 2011). Thus, most studies support the idea that signal transmission in the TGF starts with a regulated release of ATP from macula densa cells and ends with constriction of afferent arterioles by adenosine via A_1_ receptors.

One of the ways that the TGF system can be studied is through micropuncture experiments. The stop-flow pressure in the proximal tubule upstream to a wax block is a relative index of glomerular capillary pressure. Stop-flow pressure changes while perfusing the distal nephron, distal from the wax block, with an artificial tubular fluid at different flow rates. There will be a progressive drop in glomerular capillary pressure when flow is increased above a certain level as indicated in the control curve in the Figure 1B. Both elevated angiotensin II levels and increased formation of reactive oxygen species (ROS) may sensitize the TGF system, i.e., to be activated at a lower flow rate and respond with a larger reduction in glomerular capillary pressure. Angiotensin II itself can increase the generation of superoxide by activation of the NADPH oxidase, which strengthens the TGF. On the other hand, it also releases NO that desensitizes the TGF system to reduce the TGF response (Liu and Persson, 2004; Patzak et al., 2004). During desensitization, TGF occurs then at a higher tubular flow rate than normal and with a smaller response. An important question that has been debated is how this modulation of TGF by angiotensin II, ROS, and NO can occur and what are the underlying mechanisms(s). The AA is the effector site of TGF, and through microperfusion of isolated afferent arterioles we have determined the contractile responses to angiotensin II and adenosine alone, and their interaction. In one series of experiments, increasing concentrations of adenosine were administered. Figure 2A shows a modest arteriolar contraction (7%) to adenosine in low concentrations, whereas high adenosine concentrations induce vasodilation. The dilatory response in the high concentration range is mediated by activation of adenosine A_2_ receptors (Lai et al., 2006). In the presence of angiotensin II in low concentrations, the arteriolar response to cumulative applications of adenosine was significantly enhanced (Figure 2A). Bolus application of angiotensin II alone at 10^−12^ and 10^−10^ mol/L induced negligible vasoconstrictions (Lai et al., 2009). Therefore, it is clear that the addition of angiotensin II sensitizes the contractile response to increased concentrations of adenosine.

Nonselective inhibition of nitric oxide synthase (NOS) with L-NAME further amplified the contractile response when added to the combined solution of angiotensin II (10^−10^ mol/l) and increasing concentrations of adenosine (Figure 2B). In contrast, the NO donor spermine NONOate (5 × 10^−4^ mol/l) completely abolished the contractile response to angiotensin II and adenosine (Figure 2B).

**Figure 2:**
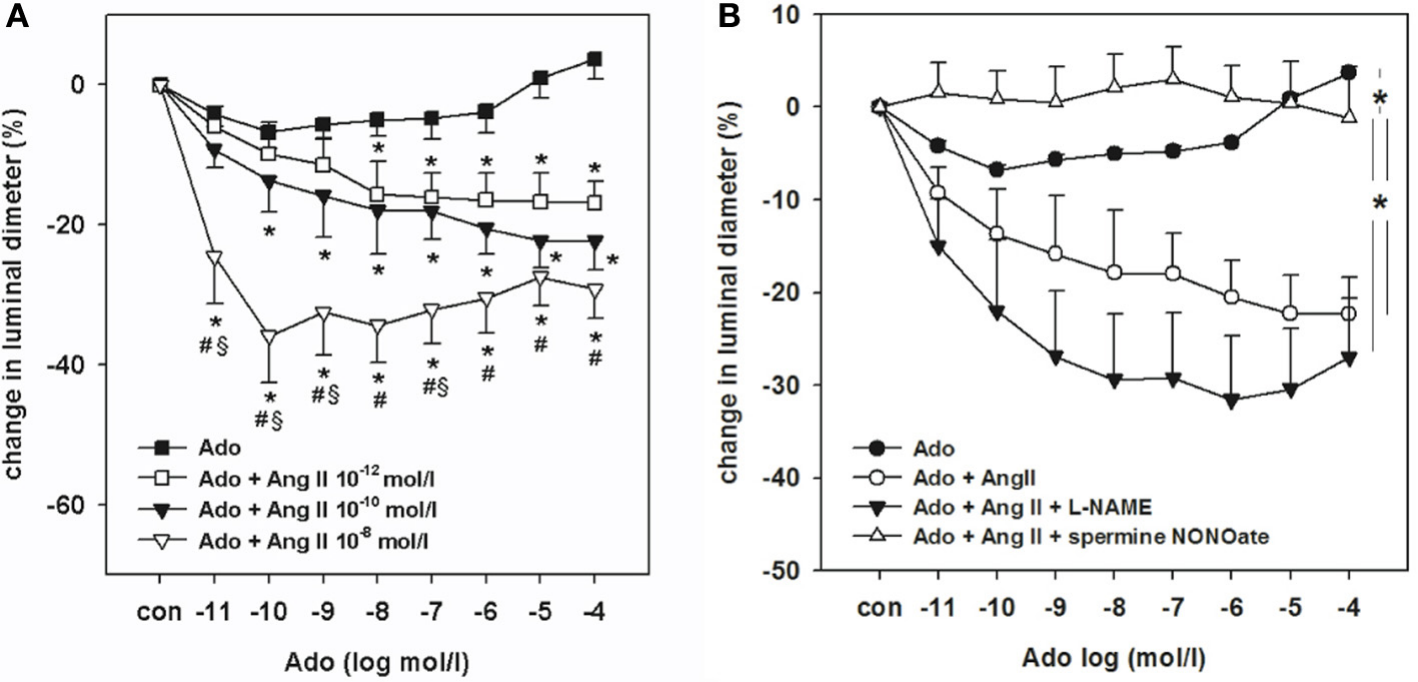
**(A)** Dose response curve for adenosine on afferent arteriolar contraction in increasing concentration from 10^−12^ to 10^−4^ mol/lwith and without the presence of angiotensin II in three different doses [10^−12^, 10^−10^, 10^−8^ mol/l, from Lai et al. (2009)] **(B)** Dose response curve for adenosine in increasing concentrations alone or together with angiotensin II (10^−10^ mol/l), or angiotensin II (10^−10^ mol/l) + L-NAME (10^−4^ mol/l) or angiotensin II (10^−10^ mol/l) + L-NAME (10^−4^ mol/l) + spermine-NONOate (5 × 10^−4^ mol/l) a NO donor. ^*^indicates differences in the course of concentration response curves.

Comparing the TGF response curve with the contractions curves from the AA is very interesting. With the present understanding of how the macula densa signal is transmitted we would expect an increased release of adenosine on an increased NaCl load to this segment. The contractile response of the AA will then depend on individual concentrations of angiotensin II, ROS and NO in the juxtaglomerular apparatus. The arteriolar data tells us that if angiotensin II concentration is high there will be a larger response at a lower release of adenosine explaining the increase in TGF sensitivity. On the other hand in a situation with a lot of NO released it will act to reduce the contraction and thereby shifting the TGF sensitivity to a less sensitive level.

We suggest that these interactions of vasoactive substances on the afferent arteriolar contractions can explain at least a part of the phenomenon of resetting of the TGF by angiotensin II, ROS and NO. Increased arteriolar reactivity and TGF responses have been described in several models for hypertension, which may be associated to the abnormal regulation of renal angiotensin II *(elevated)*, oxidative stress *(increased)* and NO bioavailability *(reduced).*

## Conflict of interest statement

The authors declare that the research was conducted in the absence of any commercial or financial relationships that could be construed as a potential conflict of interest.
